# Effective bias warm-up time reduction for MEMS gyroscopes based on active suppression of the coupling stiffness

**DOI:** 10.1038/s41378-019-0057-2

**Published:** 2019-05-06

**Authors:** Jian Cui, Qiancheng Zhao, Guizhen Yan

**Affiliations:** The National Key Laboratory of Science and Technology on Micro/Nano Fabrication, Institute of Microelectronics, Peking University, 100871 Beijing, China

**Keywords:** Electrical and electronic engineering, Sensors

## Abstract

Bias warm-up time is the time required for MEMS gyroscopes to reach a relatively stable state with specified performance after the power supply is turned on, is a critical factor for short time-of-flight navigation applications. This paper demonstrates an effective method to improve the bias warm-up time of a custom-designed MEMS gyroscope operating in split-mode based on open-loop readout scheme with active suppression of the coupling stiffness (ASCS). A semi-quantitative mathematical model for the temperature sensitivity of the bias is established that indicates the resonant frequency of the primary mode, the frequency difference and the coupling stiffness between the primary and sense modes together with the demodulation phase error, and these are the main factors that affect bias warm-up time. Of all these parameters, the stiffness coupling variation contributes the most to the start-up warm-up time, followed by the phase error drift. The experimental result shows that the bias warm-up time decreases from 2000 to 2 s under the condition that the bias stability (1*σ*) falls into about 10 deg/h within an hour of testing time using closed-loop control for the coupling stiffness, resulting in a reduction of three orders of magnitude. In addition, the bias instability of the gyroscope is improved two-fold from 4 to 2 deg/h with ASCS.

## MEMS: Fast Start-up gyroscopes

A method to achieve reduced bias warm-up time in MEMS gyroscopes could lead to enhanced technologies. MEMS gyroscopes are widely used in tactical applications, including gun-launched projectiles and tactical missile guidance. However, such applications require that the bias warm-up time – the time period between turning on the gyroscope and it achieving a stable state – is as short as possible; for example, one can imagine that a projectile will only be in-flight for a matter of seconds in some instances, and achieving stability in the gyroscope as soon as possible is vital. Now, a team led by Guizhen Yan from Institute of Microelectronics, Peking University, demonstrates that suppressing the coupling stiffness provides a simple and cost-effective way to reduce the bias warm-up time, achieving a three order of magnitude reduction.

## Introduction

The consumer electronic products represented by the smart phones have witnessed the great success of MEMS gyroscopes in the past few years due to their low-cost, miniature size, and light weight. The performance of MEMS instruments is continually improving, accompanied by the rapid progress of fabrication and electronics technology, and this will push the development of these microsensors for military applications including tactical-grade and even navigation-grade missions^1–3^. It is expected that the tactical performance end of the inertial sensor application spectrum will likely be dominated by MEMS gyroscopes, such as gun-launched projectiles, tactical missile guidance, fire control systems, radar antenna motion stabilization, and so on^4,5^. The remarkable feature of tactical-grade applications is fast. For instance, the majority of missile missions are <180 s (3 min) duration^6^. The in-flight time of artillery and tank applications are only 200 and 10 s^7^. These short time-of-flight and rapid reaction weapons put stringent requirements on the gyroscope bias start-up time when the power supply is turned on. Allan variance is preferred to investigate the sensor error behavior on different timescales for academic purposes, of which the bias instability caused by random flicker noise is normally used to evaluate the bias performance of the gyroscopes. However, this performance index cannot be the basis for selection of MEMS gyroscopes in practical tactical applications. Instead, the bias stability (1*σ*) can reflect the sensor output overall fluctuation over a specified time of continuous operation. In practical applications, what the customers are most concerned about is how long it takes for the bias output of the gyroscope to enter a stable status. This characteristic can be reflected by the bias warm-up time defined as the time from the initial application of power for a sensor to reach specified bias stability under specified operating conditions^8^.

When the power supply is turned on, the gyroscope experiences a slight temperature rise due to heat transferred from the peripheral readout circuit, which can result in variations of the package stress and structure resonant frequencies and thus the output bias drift. The key to understanding bias thermal drift is to find the main components affecting bias output. The error sources defining tactical-grade performance in silicon tuning-fork gyroscopes are first analyzed in ref. ^9^ including mechanical quadrature, fluid coupling, and electrical coupling. Five bias error sources for one particular MEMS gyroscope implementation are studied experimentally and quantitatively identified^10^, which show that cross-coupling in the structure and electronics are the two most important sources in their implementation. Similar work is also found in refs. ^11,12^ that establishes a bias contribution model for a dual mass tuning fork structure and single-mass structure, respectively, in which the bias can be classified into two categories of in-phase and quadrature error. Although the bias error sources are studied in detail from the work mentioned above, few literature studies have reported on the bias warm-up time behavior. The bias-time characteristics during the thermal start-up process are modeled by analyses of different transient responses via different pathways in the heat transfer process^13^. However, an effective method to shorten the warm-up time is not validated. The temperature self-sensing method is used to improve bias drift from 2000 to 5 deg/h during a thermal start-up time of 900 s without using external temperature sensors and considering the drive mode resonant frequency to be an embedded thermometer^14^. On-chip temperature control can be used to realize fast start-up by heating the sensor structure to high temperature quickly^15,16^. Nevertheless, this method increases the system power consumption, as well as the mechanical and electrical thermal noise.

In this paper, we focus on reducing the bias warm-up time of the MEMS gyroscope for tactical applications that are cost sensitive. Although the mode-matched gyroscope is normally considered a very effective technical solution to obtain high performance^17–20^, the complexity of the readout circuit and the device cost inevitably increase in that at least four control loops are necessary: the drive, quadrature control, rate rebalance and mode matching loops. In some cases, passive post-trimming methods, such as laser trimming^21^, focused ion beam trimming^22^, and mass loading^23^ are required for extremely small and stable frequency split. This type of tuning is inflexible, time consuming, and therefore also significantly adds to the gyroscope cost. Compared to the mode-matched gyroscope, the circuit of the split-mode type with open-loop readout is simple, leading to low-cost batch production for tactical applications. Thus, the split-mode gyroscope is selected as the object in this study. The main factors with their influences on bias warm-up time are theoretically and experimentally investigated. We found that the stiffness coupling variation contributed the most to bias warm-up time, followed by phase error drift for the silicon tuning fork gyroscope designed by our group. Therefore, to reduce the bias warm-up time, active suppression of the quadrature error is applied to the custom-designed gyroscope operating in split-mode by adjusting the coupling stiffness in closed-loop control fashion. By doing so, we not only significantly decrease the bias warm-up time but also improve the bias instability of the gyroscope.

## Materials and methods

### Bias temperature characteristics during warm-up time

**Fig. 1:**
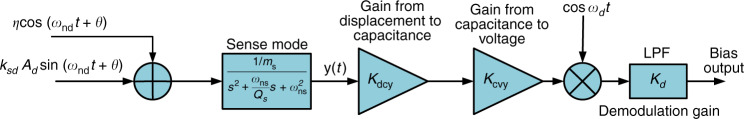
Schematic of the open-loop readout signal chain of the angular rate for sense mode

Figure 1 shows the open-loop readout signal chain of the angular rate for the sense mode schematic, which is a prevailing configuration used in MEMS gyroscopes. The vibration displacement of the proof mass *y*(*t*) caused by external forces is first converted to capacitance variation and then to voltage by a low noise pre-readout circuit with the gain of *K*_*dcy*_*,K*_*cvy*_, respectively. A phase sensitive synchronous demodulator with a low-pass filter is used to extract the angular rate signal from the vibration signal of sense mode.

Assuming that the drive voltage is *F*_drv_(*t*) = *F*_el_ cos *ω*_nd_*t*, the vibration displacement of the proof mass is written by

1 $$x(t) = A_{{d}}\sin (\omega _{\rm{{{nd}}}}t + \theta )$$

where *ω*_nd_ is the resonant frequency of the drive mode and *θ* is the phase error caused by the phase shift circuit in the drive loop circuit.

Then, the external force exerted on the sense mode is given by

2 $$F_{{s}}(t) = k_{\rm{{{sd}}}}A_{{d}}\sin (\omega _{\rm{{{nd}}}}t + \theta ) + \eta \cos (\omega _{\rm{{{nd}}}}t + \theta )$$

where *k*_sd_ is the coupling stiffness from the drive to sense mode and *η* is the amplitude of equivalent in-phase force including damping and electrical coupling.

The magnitude and phase response of the sense mode dynamics operating in split mode with a high-quality factor at the resonant frequency is written as

3 $$\begin{array}{c}\left| {G_{{s}}(j\omega _{{\rm{nd}}})} \right| = \frac{{1/m_{{s}}}}{{\sqrt {\left[ {\omega _{\rm{{{ns}}}}^2 - \omega _{\rm{{{nd}}}}^2} \right]^2 + \frac{1}{{Q_{{s}}^2}}[\omega _{\rm{{{ns}}}}\omega _{\rm{{{nd}}}}]^2} }}\\ = \approx \frac{1}{{m_{{s}}\omega _{\rm{{{ns}}}}^2}}\frac{1}{{1 - (\frac{{\omega _{\rm{{{nd}}}}}}{{\omega _{\rm{{{ns}}}}}})^2}} = \frac{{1/m_{{s}}}}{{\omega _{\rm{{{ns}}}}^2 - \omega _{\rm{{{nd}}}}^2}}\end{array}$$

4 $$\angle G_{{s}}(j\omega _{\rm{{{nd}}}}) = \beta \left( {j\omega _{\rm{{{nd}}}}} \right) = - {\rm{atan}}\frac{{\eta _{{f}}Q_y^{ - 1}}}{{1 - \eta _{{f}}^2}},\eta _{{f}} = \frac{{\omega _{\rm{{{nd}}}}}}{{\omega _{\rm{{{ns}}}}}}$$

where *m*_s_ is the effective sense proof mass and *Q*_s_ is the sense mode quality factor. Thus, the bias output is obtained by multiplying the vibration signal due to external forces by the drive signal and filtering the high-frequency component

5 $$\begin{array}{c}V_{{o}}(t) = \left\{ {k_{\rm{{{sd}}}}A_{{d}}\sin [\theta + \beta (j\omega _{\rm{{{nd}}}})]} \right.\\ \left. { + \eta \cos [\theta + \beta (j\omega _{\rm{{{nd}}}})]} \right\}K_{\rm{{dcy}}}K_{\rm{cvy}}K_{{d}}\left| {G_{{s}}(j\omega _{\rm{{{nd}}}})} \right|\end{array}$$

Self-oscillation with automatic gain control is used to drive the proof mass at its resonant frequency and keep constant vibration amplitude of *x*(*t*). Thus, we get

6 $$A_{{d}}K_{\rm{dcx}}K_{\rm{cvx}} = R$$

where *K*_dcx_*,K*_cvx_, are the gains from displacement to capacitance and from capacitance to voltage for drive mode, respectively. *R* is the control target voltage produced by a reference. Substituting Eqs. (3) and (6) into Eq. (5), the bias output can be obtained as

7 $$\begin{array}{c}{\rm{{Bias}}} \approx \frac{{K_{\rm{dcy}}K_{\rm{cvy}}}}{{K_{\rm{dcx}}K_{\rm{cvx}}}}\frac{{k_{\rm{{{sd}}}}RK_{{d}}}}{{2m_{{s}}\omega _{\rm{{{nd}}}}\Delta \omega }}\sin (\theta {{ + }}\beta ){{ + }}\eta \cos (\theta {{ + }}\beta )\\ \approx \frac{{K_{\rm{dcy}}K_{\rm{cvy}}}}{{K_{\rm{dcx}}K_{\rm{cvx}}}}\frac{{k_{\rm{{{sd}}}}RK_{{d}}}}{{2m_{{s}}\omega _{\rm{{{nd}}}}\Delta \omega }}\sin (\theta {{ + }}\beta )\end{array}$$

where Δ*ω* is the frequency split of the two working modes. Thanks to the high vacuum gyroscope packaging, the in-phase component caused by fluid and electrical coupling can be neglected^9^. When the power is turned on, the heat generated by the peripheral circuit will be partially transferred to the gyroscope structure through the package pins and sensor substrate, resulting in rising temperature. The heat transferred through the substrate is dominant^13^. This means that the warm-up time is determined by the bias thermal drift, which can be described by the temperature sensitivity. The temperature sensitivity is expressed as the derivative of the bias with respect to temperature as follows:

$$\begin{array}{c}\frac{1}{{\rm{{{Bias}}}}}\frac{{\partial {\rm{{Bias}}}}}{{\partial T}} =	 \hskip -20pt \frac{1}{{k_{\rm{{{sd}}}}}}\frac{{\partial k_{\rm{{{sd}}}}}}{{\partial T}} + \frac{1}{R}\frac{{\partial R}}{{\partial T}} + \frac{1}{{K_{{d}}}}\frac{{\partial K_{{d}}}}{{\partial T}} + \frac{1}{{K_{dcy}}}\frac{{\partial K_{dcy}}}{{\partial T}}\\ 	\hskip -30pt - \frac{1}{{K_{\rm{{dcx}}}}}\frac{{\partial K_{\rm{dcx}}}}{{\partial T}} + \frac{1}{{K_{\rm{cvy}}}}\frac{{\partial K_{\rm{cvy}}}}{{\partial T}} - \frac{1}{{K_{\rm{cvx}}}}\frac{{\partial K_{\rm{cvx}}}}{{\partial T}}\\ 	\hskip -10pt - \frac{1}{{\omega _{\rm{{{nd}}}}}}\frac{{\partial \omega _{\rm{{{nd}}}}}}{{\partial T}} - \frac{1}{{\Delta \omega }}\frac{{\partial \Delta \omega }}{{\partial T}} + \frac{1}{{\sin \left[ {\theta + \beta } \right]}}\frac{{\partial \sin [\theta + \beta ]}}{{\partial T}}\end{array}$$

The geometric dimension variations of the detection sense combs in drive and sense modes are so similar when the temperature changes that the effect brought by *K*_*dcx*_ and *K*_*dcy*_ can be largely reduced. To attenuate the drift of the pre-readout circuit gain, the readout circuits of the primary and the sense modes are designed to be the same. The demodulation gain *K*_d_ is configured by a second-order Butterworth low-pass filter composed of operational amplifiers, capacitors, and resistors and is equal to the ratio of two resistors. The temperature coefficients (TCFs) of the resistors are very low and close, ~5 ppm/°C. Thus, the thermal drift of *K*_d_ can be omitted. For the temperature drift of *R*, an ultraprecision, low noise voltage reference ADR420 is employed with an ~1 ppm/°C TCF. Therefore, the effect of *R* on the bias is also ignored. Eq. (7) can be simplified as follows:

8 $$\begin{array}{c}\frac{1}{{\rm{{{Bias}}}}}\frac{{\partial {\rm{{Bias}}}}}{{\partial T}} \approx \frac{1}{{k_{\rm{{{sd}}}}}}\frac{{\partial k_{\rm{{{sd}}}}}}{{\partial T}} - \frac{1}{{\omega _{\rm{{{nd}}}}}}\frac{{\partial \omega _{\rm{{{nd}}}}}}{{\partial T}}\\ - \frac{1}{{\Delta \omega }}\frac{{\partial \Delta \omega }}{{\partial T}} + \frac{1}{{\sin \left[ {\theta + \beta } \right]}}\frac{{\partial \sin [\theta + \beta ]}}{{\partial T}}\end{array}$$

Substituting Eq. (4) into Eq. (8), the temperature sensitivity of the bias becomes

9 $$\begin{array}{c}\frac{1}{{\rm{{{Bias}}}}}\frac{{\partial {\rm{{Bias}}}}}{{\partial T}} \approx \frac{1}{{k_{\rm{{{sd}}}}}}\frac{{\partial k_{\rm{{{sd}}}}}}{{\partial T}} - \frac{1}{{\omega _{\rm{{{nd}}}}}}\frac{{\partial \omega _{\rm{{{nd}}}}}}{{\partial T}} - \frac{1}{{\Delta \omega }}\frac{{\partial \Delta \omega }}{{\partial T}}\\ + \frac{1}{{\tan \left[ {\theta + \beta (\omega _{\rm{{{nd}}}})} \right]}}\frac{{\eta _{{f}}Q_y^{ - 1}}}{{(1 - \eta _{{f}}^2)}}\frac{1}{{Q_y}}\frac{{\partial Q_y}}{{\partial T}}\\ + \frac{1}{{\tan \left[ {\theta + \beta (\omega _{\rm{{{nd}}}})} \right]}}\frac{{\partial \theta }}{{\partial T}}\end{array}$$

The sense mode only introduces about 0.1° phase delay *β* at the resonant frequency, and the temperature variation can be omitted compared with *θ* due to the high-quality factor and a relatively large frequency split for the two working modes. In addition, the demodulation phase error *θ* is usually a small quantity. Therefore, Eq. (9) can be written as

10 $$\frac{1}{{\rm{{{Bias}}}}}\frac{{\partial {\rm{{Bias}}}}}{{\partial T}} \approx \frac{1}{{k_{\rm{{{sd}}}}}}\frac{{\partial k_{\rm{{{sd}}}}}}{{\partial T}} - \frac{1}{{\omega _{\rm{{{nd}}}}}}\frac{{\partial \omega _{\rm{{{nd}}}}}}{{\partial T}} - \frac{1}{{\Delta \omega }}\frac{{\partial \Delta \omega }}{{\partial T}} + \frac{1}{\theta }\frac{{\partial \theta }}{{\partial T}}$$

Equation (10) shows that the coupling stiffness from drive to sense mode, the resonant frequency of the drive mode, and the frequency split between the two working modes together with the demodulation phase error are the main factors affecting bias thermal drift, and moreover warm-up time. The drive mode resonant frequency *ω*_nd_ and frequency split Δ*ω* are linear with ambient temperature. Their TCFs are mainly determined by Young’s modulus of the silicon material^14,24^. Thus, it is difficult to restrain the thermal variation of *ω*_nd_ and Δ*ω* with active control. However, a passive compensation method that uses *ω*_nd_ and Δ*ω* can be adopted to reduce the bias thermal drift during the start-up process, thus shortening the bias warm-up time. *ω*_nd_ can be measured by the PLL information used in the drive loop to compensate for the bias^14^. The frequency split is hard to obtain directly, which is also a key problem for mode matching control in MEMS gyroscopes. Hence, the effective means to restrain bias drift during the start-up process are decreasing the variations of coupling stiffness and the demodulation phase error, on which are focused in this study.

### Active suppression of the coupling stiffness (ASCS)

The nonideal quadrature motion is direct coupling of the drive mode displacement to the sense mode of the gyroscope due to fabrication imperfections, and it leads to nondiagonal terms in the spring matrix of the gyroscope dynamics^25^. The suppression of the coupling stiffness will decrease the quadrature error, thus improving bias drift. There are four methods reported in the literature to restrain the quadrature error. The first is to use laser trimming of the structure mass to balance structural imperfections^26,27^, which is a time-consuming task normally including coarse and fine tuning. Also, it requires complex expensive laser system equipment, inevitably increasing the cost of the sensors. The second method is pure electronic cancellation injecting charge into the preamplifier inputs^28^, which can be applied to any microgyroscope. The third approach relies on force rebalance control with actuation electrodes in sense mode^10,29^ and is usually used in gyroscopes with closed-loop readout. The drawback of the second and third methods is the need for a strictly precise phase control since they use AC signal feedback control. The fourth scheme is electromechanical coupling stiffness suppression that generates electrostatic forces inherently in phase with the displacement of drive mode to cancel the quadrature force at its origin^30,31^. The effectiveness and simple control circuits make this method superior to the other techniques and therefore the priority selection.

**Fig. 2:**
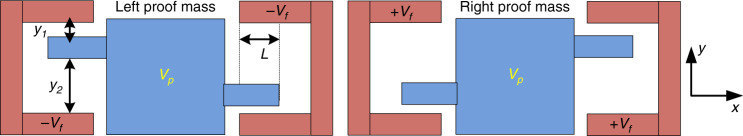
Schematic of the electrode configurations for coupling stiffness suppression

To realize electromechanical coupling stiffness suppression, several groups of comb fingers should be added to the structure proof mass as shown in Fig. 2. The tuning fork structure contains two proof masses biased with DC voltage *V*_p_. ±*V*_f_ are the DC voltages used to adjust the coupling stiffness.

Suppose Δ*x* is the displacement of the drive direction (*x*) and *N* is the number of suppressing electrodes, the electrostatic force generated in sense direction (*y*) is thus obtained as

11 $$F_y = 4N\varepsilon h(\frac{1}{{y_1^2}} - \frac{1}{{y_2^2}})V_{{p}}V_{{f}}\Delta x$$

Equation (11) shows that the phase of the generated electrostatic force is the same as the quadrature force (see Eq. (2)), avoiding the residual quadrature due to circuit phase error. The governing dynamic equation of sense mode is written as

12 $${m_{{s}}\ddot y + \frac{{m_{{s}}\omega _{\rm{{{ns}}}}}}{{Q_{{s}}}}\dot y + m_{{s}}\omega _{\rm{{{ns}}}}^2y = [4N\varepsilon h(\frac{1}{{y_1^2}} - \frac{1}{{y_2^2}})V_{{p}}V_{{f}} - k_{\rm{{{sd}}}}]\Delta x}$$

It can be seen from Eq. (12) that the coupling stiffness from drive to sense mode *k*_sd_ can be counteracted by adjusting the DC voltage *V*_f_, therefore alleviating the effect brought by the quadrature error.

### Closed-loop control for quadrature error

**Fig. 3:**
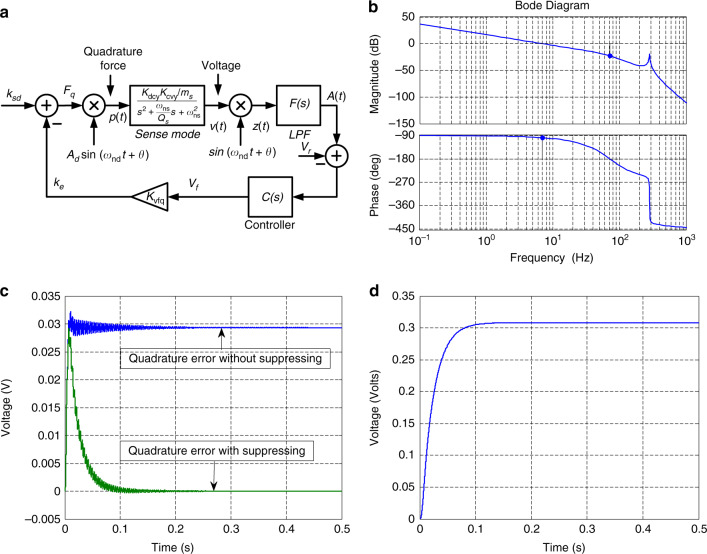
**a** Schematic of the closed-loop control for coupling stiffness. **b** Bode plot of the designed open-loop transfer function. **c** Simulation of the quadrature error voltage with and without suppression. **d** Simulation of the suppression voltage over time

Because the coupling stiffness varies with temperature, adjusting the DC voltage *V*_f_, cannot remove the quadrature error effectively during warm-up time. To tackle this problem, a closed-loop control is preferred to suppress the coupling stiffness by altering the feedback voltage according to the quadrature amplitude as shown in Fig. 3a.

The amplitude of the quadrature error *A*(*t*) is extracted by the phase demodulation followed by a second-order low-pass filter *F*(*s*). Then, the quadrature amplitude is compared to a target reference voltage *V*_r_ (here, set as zero), and the error signal is fed to a controller that is normally a PI controller to get the suppressing voltage *V*_f_. This DC voltage is differentially applied to the electrodes for coupling stiffness suppression as shown in Fig. 2. The control system is nonlinear, and it is difficult to determine the controller parameters. Thus, a baseband equivalent model for the sense mode dynamics should be established to form a linear time-invariant system^32^. The transfer function from *F*_*q*_ to *z*(*t*) can be obtained as follows:

13 $$G(s) = \frac{{K_{\rm{dcy}}K_{\rm{cvy}}A_{{d}}}}{{2\omega _{\rm{{{nd}}}}m_y}} \cdot \frac{{\Delta \omega }}{{s^2 + \frac{{\omega _{\rm{{{ns}}}}}}{{Q_{{s}}}}s + \Delta \omega ^2}}$$

Therefore, the open-loop transfer function for this system can be expressed as

14 $$L(s) = \frac{{K_{\rm{vfq}}K_{\rm{dcy}}K_{\rm{cvy}}A_{{d}}}}{{2\omega _{\rm{{{nd}}}}m_y}} \cdot \frac{{\Delta \omega }}{{s^2 + \frac{{\omega _{\rm{{{ns}}}}}}{{Q_{{s}}}}s + \Delta \omega ^2}} \cdot F(s) \cdot C(s)$$

Equation (14) is used to design the parameters of the PI controller *C*(*s*). Note that the transfer function (13) has a resonant peak at frequency Δ*ω*. The cut-off frequency of the low-pass filter should be less than Δ*ω* to attenuate the gain at frequency Δ*ω* below zero dB guaranteeing enough system amplitude margin. A PI controller is adopted to increase the gain at low frequency to reduce static error. The frequency response of the open-loop transfer function is plotted in Fig. 3b. The phase and gain margins are ~82° and 22.7 dB ensuring a robust stable control system.

The closed-loop control system model for quadrature error is constructed in the SIMULINK design environment. The parameters used in simulation are obtained by testing the real system including the gyroscope and the control circuit. Figure 3c shows the simulation of quadrature error voltage *A*(*t*) before and after suppressing the coupling stiffness. The original quadrature error is around 30 mV without any restraint. When the active suppression loop is applied, the quadrature voltage rapidly decreases to near zero within 100 ms. Meanwhile, the control voltage *V*_f_ reaches a steady state of ~300 mV as shown in Fig. 3d.

### Gyroscope design and fabrication

**Fig. 4:**
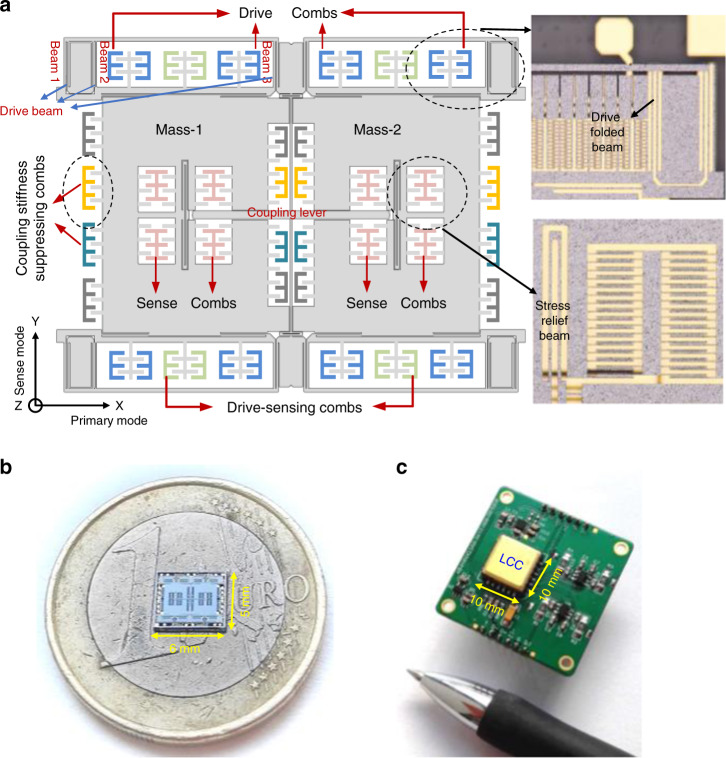
**a** Schematic and optical photos of the gyroscope. **b** Photograph of the gyroscope. **c** Testing PCB with all the control circuits and gyroscope

Figure 4a illustrates a tuning fork gyroscope designed by our group. The gyroscope has two symmetrically arranged proof masses moving parallel to the substrate in opposite directions along the *X*-axis that are connected to an outer frame fixed to anchors by four groups of folded beams. If the structure is subject to rotation around the *Z*-axis, the two proof masses will also move in opposite directions along the *Y*-axis, which is ideally orthogonal to the drive-mode direction. Although three beams whose top ends are connected through a long beam are used in each group of folded beams to restrain the displacement coupling from drive to sense mode, the spring stiffness imbalance due to beam width fabrication nonuniformity can result in rotation of the proof mass generating quadrature motion. Four groups of comb structures with electrodes are designed on the two sides of a proof mass to suppress coupling stiffness. A coupling lever is added to associate the motion of the two masses with four stress relief beams at the two ends. Unlike drive mode, squeeze film comb fingers are utilized in sense mode to achieve high mechanical sensitivity. The resonant frequencies of drive mode and the frequency split are designed to be ~10 kHz and 300 Hz, which are close to the measurement results of 10,429 and 276 Hz.

The gyroscope is fabricated with a silicon on glass (SOG) process using silicon/glass wafer bonding and deep reactive ion etching (DRIE). First, the regions for the anchors and the mechanical moving structures are defined by 30-µm DRIE etching. An ~200-nm Ti/Pt/Au layer is sputtered on the glass wafer and patterned by a lift-off process to form a metal interconnection electrode. Next, the two wafers are anodically bonded together and the silicon wafer is thinned to about 110 µm by KOH etching for 80-µm-thick device structures. Finally, the gyroscope structure is released by DRIE etching. The size of the gyroscope chip is 6 × 6 mm as shown in Fig. 4b.

To enhance the signal-to-noise ratio and decrease the phase delay *β* caused by the sense mode dynamics regarding the resonant frequency of the primary mode, the gyroscope is vacuum sealed in a custom-designed LCC packaging. A gold-plated lid is sputtered on the inside surface with a thin film getter made of titanium to maintain a high vacuum inside the sealed cavity. The quality factors of the primary and sense modes are measured to be ~16,000 and 9800, respectively. To test the bias performance of the gyroscope, a pure analog circuit is designed with discrete components including closed-loop driving and synchronous demodulation. A PCB is fabricated to mount with the test circuit, and the gyroscope is vacuum sealed in an LCC package as shown in Fig. 4c. The key gyroscope parameters are listed in Table 1.

**Table 1:** The key gyroscope parameters

Parameter	Value
Drive frequency	10,429 Hz
Sense frequency	10,705 Hz
Quality factor of drive mode	16,000
Quality factor of sense mode	9800
Structure thickness	80 μm
Sense capacitance of drive mode	0.6 pF
Sense capacitance of sense mode	2.3 pF

## Results and discussion

A PXI4462 four-channel data acquisition card is adopted to acquire the gyroscope bias output, the quadrature error amplitude, and the drive and vibration signals for drive mode. The PXI4462 resolution is 24 bits with analog and digital anti-aliasing filters embedded. The sampling rate is selected as 200 kHz to enable enough phase resolution for the detection signal. An FFT operation is applied to the drive and vibration signals of drive mode to calculate the resonant frequency *ω*_nd_ and phase error *θ*.

### Warm-up time characterization of the gyroscope without ASCS

**Fig. 5:**
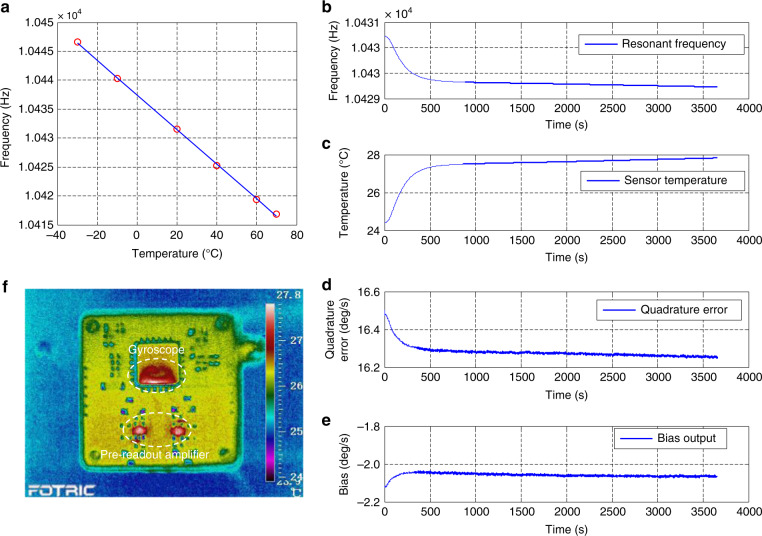
**a** Resonant frequency variation of drive mode over −30 to 70 °C. **b** Variations of resonant frequency and **c** sensor temperature when the power supply is turned on. **d** Quadrature error and **e** bias output testing when the power supply is turned on without ASCS. **f** Thermal field distribution of the test PCB on which the gyroscope is soldered

Bias stability (1*σ*) of 10 deg/h is a typical value for tactical-grade gyroscopes and is considered a criterion for measuring warm-up time. To evaluate the warm-up accurately, the power supply is turned off for 1 h before the bias testing, which lasts for an hour with a 400 Hz sampling rate and averaged to 1 Hz. The power supply is program-controlled with LabVIEW to reduce manual operation time delay. The conventional temperature sensing method using external temperature sensors suffers from thermal mismatch between the gyroscope structure and the sensor due to a temperature gradient. The drive mode resonant frequency changes linearly with the ambient temperature (see Fig. 5a) and can be viewed as a good temperature indicator. The expression for the resonant frequency and temperature is obtained by a linear fit to the frequency–temperature data. The TCF is calculated to be about −0.3 Hz/°C.

15 $$f_{{d}} = - 0.2989T + 10437.3$$

The temperature variations during 1 h of bias testing can be obtained by transforming the resonant frequency as shown in Fig. 5b to the temperature via Eq. (15) as shown in Fig. 5c. The temperature of the gyroscope structure rises rapidly within 500 s and reaches a stable point at about 1000 s. The variation range of the resonant frequency can be calculated from the power on to a relative stable status as about 1 Hz corresponding to a temperature variation of about 3.4 °C. Therefore, the warm-up time is determined by bias drift during this slight temperature rising process. An infrared thermal imaging camera, Fortric226, is used to capture the thermal field distribution of the test PCB on which the gyroscope is soldered as shown in Fig. 4c. The thermal field distribution of the PCB at 3600 s is shown in Fig. 5f. The final temperature of the gyroscope is around 28 °C when the system enters thermal balance, which is very close to the calculated result shown in Fig. 5c.

Figure 5d, e present the gyroscope quadrature error and the bias output when the power supply is turned on. There is a greater correlation between the variation trends of the bias and quadrature as expected since the quadrature error can leak to zero bias through phase error (see Eq. (7)). During the first 400 s, the bias rises from the minimum to the maximum value with thermal drift of 317  deg/h. After that, the bias gradually enters a steady state. The stability (1*σ*) of the bias and the quadrature error calculated from the starting point are about 36 and 118 deg/h, respectively. It seems that the bias output is stable after 1000 s. However, the bias stability is 14.6  deg/h even from 1200 s to the end, which is larger than our specified bias performance of 10 deg/h. The warm-up time is about 2000 s, from which the bias stability is 10.2  deg/h. It is too long for tactical-grade applications.

### Quadrature nulling based on closed-loop ASCS

**Fig. 6:**
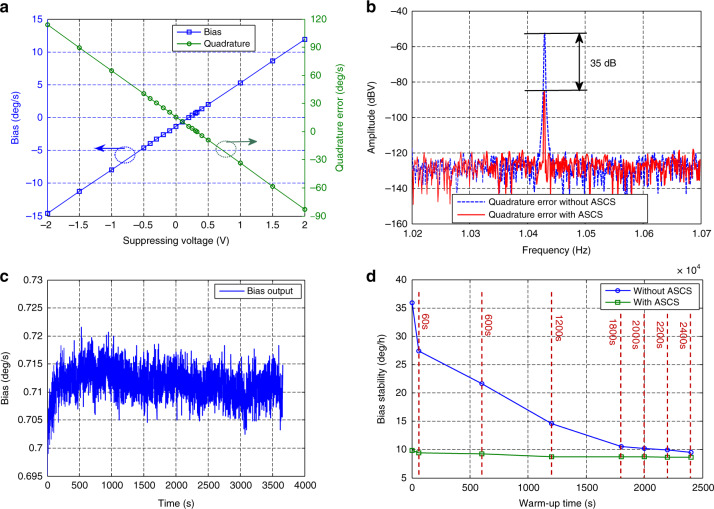
**a** The quadrature error amplitude and bias under different suppressing voltages. **b** The quadrature error with and without coupling stiffness suppression. **c** Bias output testing when the power supply is turned on with ASCS. **d** Bias stability vs. warm up time with and without ASCS

The bias performance is largely affected by the quadrature error drift as shown in Fig. 5. Therefore, the closed-loop ASCS illustrated in Fig. 3a is implemented to restrain the quadrature error and improve bias stability. To verify the feasibility of the added structure for suppressing the coupling stiffness, a series of DC voltages are applied to the suppression structure electrode. Figure 6a displays the amplitude of quadrature error and bias output under different suppressing voltages. The quadrature error varies linearly with the suppressing voltage, which proves that the coupling stiffness can be effectively adjusted using additional restraining structures. The minimum absolute value for the quadrature error appears at 325 mV, which is close to the simulation result of 300 mV. Meanwhile, the bias output almost reaches the absolute value minimum.

Figure 6b shows the pre-readout circuit spectrum for sense mode with and without closed-loop ASCS. The peak value is located at the resonant frequency of 10,429 Hz, which reflects the quadrature error amplitude. The amplitude of the coupling signal decreases by ~35 dB using closed-loop ASCS, reducing the average absolute bias value over an hour of testing time from −2.07  to 0.72 deg/s.

### Warm-up time characterization of the gyroscope with ASCS

The bias output with closed-loop ASCS is shown in Fig. 6c. The bias stability calculated from the starting point is about 9.89 deg/h, which is within 10 deg/h. Note that the bias output is not thermally compensated. Thus, the gyroscope warm-up time with ASCS is considered to be within 2 s, which is improved by three orders of magnitude compared to the 2000 s obtained without coupling stiffness suppression. Figure 6d illustrates the bias stability under different warm-up times with and without ASCS calculated from Figs. 6c and 5e. The gyroscope without coupling stiffness restraint needs a relatively long warm-up time to reach steady state. If the ASCS is adopted, the bias output can quickly achieve a stable status meeting the 10 deg/h bias stability requirements from the starting point.

**Fig. 7:**
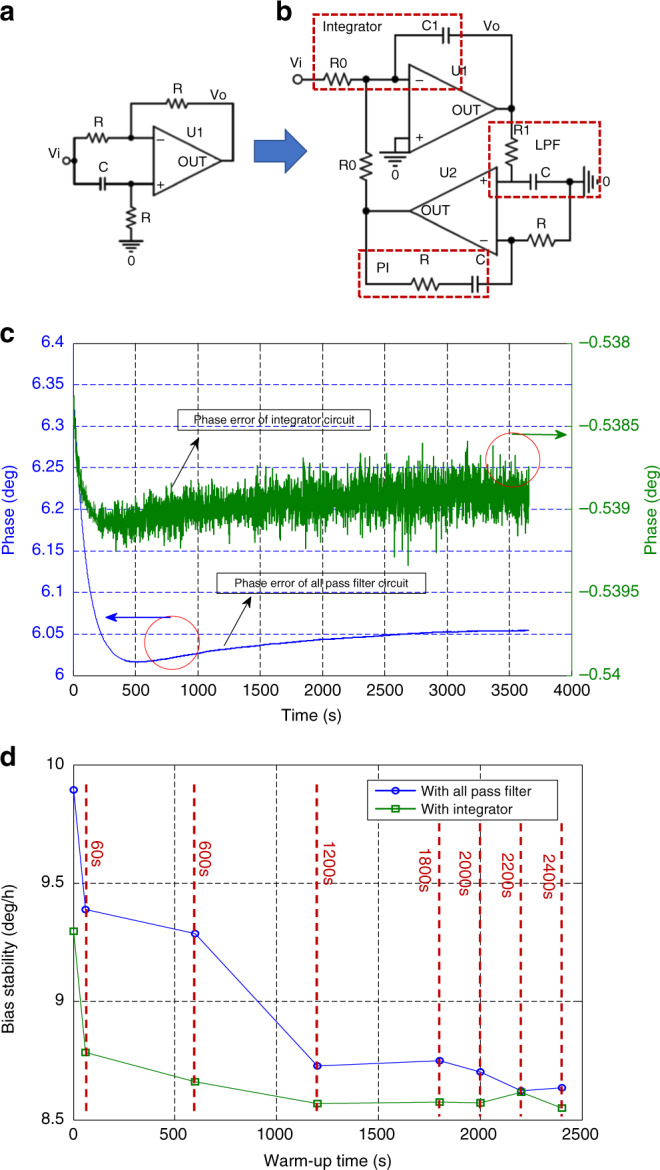
**a** All pass filter as the phase shift circuit. **b** Integrator with DC bias self-cancellation as the phase shift circuit. **c** Phase error over time of two types of phase shift circuits. **d** Bias stability vs. warm up time with two types of phase shift circuits

Another factor influencing the warm-up time is the demodulation phase error as explained in Eq. (10). The effect on the bias drift brought by the phase error is also investigated in this work. The source of the phase error mainly comes from the phase shift circuit used in the drive loop circuit for drive mode in our design. The phase shift circuit is a simple first-order all pass filter as shown in Fig. 7a with the phase response: *π*−2tan^−1^*RCω*_nd_. The temperature variation during the start-up process can alter the resistor *R*, capacitor *C*, and resonant frequency *ω*_nd_, which inevitably causes bias drift. To restrain the all pass filter phase drift, an integrator is adopted to realize quadrature phase shift. The integrator circuit normally suffers from the saturation of the output due to the continuous integrating of the minute leakage current from the operation amplifier. Thus, we utilize an integrator with a DC bias self-cancellation circuit that incorporates a low-pass filter and a PI controller to avoid the accumulation of leakage current as shown in Fig. 7b. The variations of *R*_0_ and *C*_1_ change the gain of the new phase shift circuit but not the phase, which improves the phase error stability. Figure 7c shows the phase variations of the two types of phase shift circuits with ASCS when the power is turned on. Thanks to the integrator phase shift affected by the peripheral resistor and the capacitor being very small, the stability (1*σ*) of the phase error caused by the integrator is just 0.1 mdeg, which is reduced more than 300 times compared with the 35.7 mdeg of the all pass filter circuit. However, the bias drift and the warm-up time do not improve much as expected, as shown in Fig. 7d, which demonstrates that the warm-up time is 1–2 s with ASCS whichever phase shift circuit is used because under these two conditions, bias stability is reached within 10 deg/h calculated from the starting point. It can be inferred that the stiffness coupling variation contributes the most to the start-up warm-up time, followed by phase error drift.

**Fig. 8:**
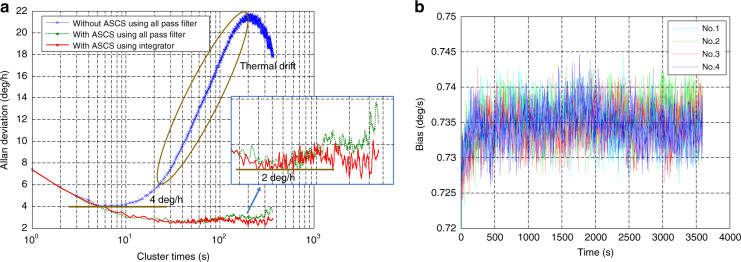
**a** Allan deviation of the gyroscope without ASCS using an all pass filter, with ASCS using an all pass filter, and with ASCS using an integrator. **b** Repeatability test of the bias output with ASCS using an integrator for a single gyroscope G01

Allan deviations of the bias output without ASCS using an all pass filter, with ASCS using an all pass filter and with ASCS using an integrator with DC self-cancellation are compared in Fig. 8a. Suppressing the coupling stiffness provides significant improvement in the long-term thermal drift, which can certainly reduce sensor warm-up time. Since bias thermal drift is effectively restrained, bias instability is decreased two-fold from 4 to 2  deg/h. It can be observed from the plots that the bias temperature drift slightly drops when using an integrator as the drive loop phase shift circuit as verified in Fig. 7d. The measured results under different conditions are summarized in Table 2.

**Table 2:** Summary of measured warm-up time, ARW, and bias instability under different conditions

Test condition	Phase shift circuit	Warm-up time^1^ (s)	ARW ( deg/h/rtHz)	Bias instability ( deg/h)
Without ASCS	All pass filter	2000	7.43	4
With ASCS	All pass filter	2	7.38	2.23
With ASCS	Integrator with DC self-cancellation	2	7.31	2.15

^1^*Note*: 10 deg/h bias stability (1*σ*) is considered the criterion for measuring warm-up time

Start-up drift experiment repeatability is important for verifying the feasibility and effectiveness of the ASCS method. This is because the environmental factors (e.g., temperature) are not the same at different starting times. Thus, we also completed the bias output repeatability test with ASCS using an integrator for a single gyroscope G01 as shown in Fig. 8b. The bias output is tested four times with a one-hour power off interval for each test to guarantee that the gyroscope temperature is the same as ambient temperature. The test results are shown in Table 3 and indicate good repeatability for the average bias value of about 1.43  deg/h (standard deviation of the four average bias values). The bias stability (1*σ*) of all four tests falls into the 10 deg/h target value, resulting in a warm-up time of about 2 s. We remove the first data due to data acquisition card error. Three gyroscope samples including G01 are selected to evaluate the effectiveness further as listed in Table 4. Thanks to the ASCS method, the warm-up time for each device is 2 s with bias stability within 10 deg/h. It should be noted that all the bias tests are without any temperature compensation. We believe that the combination of the ASCS method and thermal compensation can further improve gyroscope bias performance.

**Table 3:** Summary of the bias output repeatability test with ASCS using an integrator phase shift circuit (sample device G01)

Test time	Average bias (deg/s, 1-h data)	Bias stability (1*σ*) (deg/h, 1-h data)	Warm-up time (s)
1	0.7348	9.31	2
2	0.7353	9.48	2
3	0.7344	9.37	2
4	0.7346	9.43	2

**Table 4:** The warm-up time test of four different gyroscope samples with ASCS using an integrator phase shift circuit

Sample	Bias stability (1*σ*) (deg/h, 1-h data)	Warm-up time (s)
G01	9.31	2
G02	7.36	2
G03	8.67	2

## Conclusion

In this paper, we focus on improving gyroscope bias warm-up time, which is a crucial performance parameter for short time-of-flight and rapid reaction weapons applications. A mathematical model for bias temperature sensitivity is established that indicates the resonant frequency of the primary mode, the frequency split and the coupling stiffness between the primary and sense modes together with the demodulation phase error, and these are the main factors affecting bias warm-up time. The resonant frequency variation depends on Young’s modulus of the silicon material. Thus, an effective method to reduce warm-up time is restraining the drift of coupling stiffness and demodulation phase error. Thanks to the closed-loop ASCS, the bias warm-up time decreases from 2000 to 2 s under the condition that bias stability (1*σ*) falls into about 10  deg/h within 1 h of testing time, resulting in a reduction of three orders of magnitude. The bias instability of the gyroscope is improved two-fold from 4 to 2 deg/h with ASCS. The experimental results indicate that the stiffness coupling variation contributes the most to start-up warm-up time, followed by phase error drift.
